# Study protocol: evaluation of the probiotic *Lactobacillus Fermentum* CECT5716 for the prevention of mastitis in breastfeeding women: a randomised controlled trial

**DOI:** 10.1186/s12884-017-1330-8

**Published:** 2017-05-19

**Authors:** Diana M. Bond, Jonathan M. Morris, Natasha Nassar

**Affiliations:** 1 Clinical and Population Perinatal Health Research, Kolling Institute of Medical Research, University of Sydney, Level 2, Bldg 52, Royal North Shore Hospital Campus, St. Leonards, NSW 2065 Australia; 2 Menzies Centre for Health Policy, Charles Perkins Centre D17, The University of Sydney, Camperdown, NSW 2006 Australia; 3 Sydney Medical School – Northern, The University of Sydney, Kolling Building, Level 7, Royal North Shore Hospital, St Leonards, NSW 2065, Australia

**Keywords:** Mastitis, Probiotics, Prevention, Protocol, Randomised controlled trial, Breastfeeding

## Abstract

**Background:**

Mastitis and accompanying pain have been associated with the cessation of breastfeeding. Mastitis is an inflammatory condition of the breast and may be a result of decreased immunity and a lowered resistance to infection. Mastitis affects up to one in five breastfeeding women with most episodes occurring in the first 6–8 weeks postpartum. Antibiotics are often used in the treatment of mastitis, but have not been popular or proven effective as a preventative agent. The WHO has highlighted significant concerns relating to adverse harms of antibiotic use with the production of antibiotic-resistant strains of disease organisms. Increasing research suggests that specific probiotic bacteria possess significant anti-inflammatory properties and supports their potential use as immunomodulatory agents. While animal studies have shown promising results in the use of probiotics for preventing mastitis, their use in human trials has had limited investigation. The aim of this study is to evaluate the effectiveness of oral probiotics for the prevention of mastitis in breastfeeding women.

**Methods:**

APProve (C**A**n **P**robiotics Im**Prove** Breastfeeding Outcomes?) is a double-blind randomised controlled trial designed to assess outcomes between breastfeeding women ingesting a probiotic versus a placebo daily for 8 weeks following birth. A total of 600 women (300 to each arm) who intend to breastfeed will be randomised after the birth of a term, healthy infant. Daily and weekly surveys for 8 weeks and follow-up surveys at 2, 6 and 12 months after birth will assess the primary outcome of mastitis in the first 8 weeks following birth as well as secondary maternal outcomes of breastfeeding duration (total/partial), antibiotic use, maternal health and well-being, and treatment compliance; and infant outcomes including gastroenteritis, infant health and well-being and growth and development. The acceptability and compliance using a novel mobile phone application system will also be evaluated.

**Discussion:**

There is an urgent need to explore safe and effective alternatives for preventing mastitis in breastfeeding women. This trial seeks to provide evidence for such an alternative in the form of probiotics, which may also increase breastfeeding duration, providing long-term health, cognitive and developmental benefits for children. Decreased antibiotic usage also benefits the community and health system.

**Trial registration:**

Australian New Zealand Clinical Trials Registry: ACTRN12615000923561.

Date of registration: 4th September, 2015, retrospectively registered.

**Electronic supplementary material:**

The online version of this article (doi:10.1186/s12884-017-1330-8) contains supplementary material, which is available to authorized users.

## Background

Breastfeeding has been consistently shown to be protective against a broad range of immediate and long term infant and maternal health outcomes [1–3]. International guidelines recommend exclusive breastfeeding for all babies in the first 6 months of life as it provides the best nutritional start for infants and promotes their healthy growth and development. Given the positive benefits of breastfeeding, it is of concern that the 2010 Australian Institute of Health and Welfare (AIHW) National Health Survey reported less than 15% of women exclusively breastfeeding at 5 months of age, despite a 96% initiation rate at birth [4]. These findings suggest that more resources and research are required to identify and treat the underlying causes of breastfeeding cessation in order to extend the overall duration and improve long term maternal and child health outcomes.

Mastitis and the pain associated with the condition have been shown to be associated with the cessation of breastfeeding [5–7]. The World Health Organisation (WHO) defines mastitis as an inflammatory condition of the breast, which may or may not be accompanied by infection [8]. It occurs in 15–21% of breastfeeding mothers in Australia [9–11]. Most episodes occur in the first 6–8 weeks postpartum but can occur at any time during breastfeeding [12]. Almost one-third of women experience a recurrent episode [13–15], and up to 3% of women with mastitis develop a breast abscess, often necessitating hospitalisation [16, 17].

Little is known about the epidemiology and pathogenesis of this common condition [7]. Though not well described, the risk factors appear to be associated with milk stasis, nipple damage and maternal fatigue [8, 9]. Cracked nipples may facilitate entrance of microorganisms through the skin, and incomplete emptying of the breasts may result in milk stasis which provides a susceptible and receptive culture media for microorganisms. Decreased immunity and a lowered resistance to infection may be a result of maternal fatigue [8, 18–20]. Other risk factors include a history of mastitis with a previous child, using an antifungal nipple cream and using a manual breast pump [7, 21].

Current management of mastitis generally centers on symptom management (i.e. applying hot/cold compresses, analgesics), encouragement of the continuation of breastfeeding (including fully emptying the affected breast, feeding more frequently, and changing feeding positions often), and antibiotic therapy [7, 22]. A Cochrane review examining the effectiveness of antibiotic therapies in treating symptoms of mastitis in breastfeeding women showed a trend towards a benefit of treatment, but only 2 trials were included and were of limited quality, with one conducted over 30 years ago. As such, authors deduced there was insufficient evidence to confirm or refute the use of antibiotics in the treatment of lactational mastitis [23].

Optimal management for the prevention of mastitis is not known. A Cochrane review specifically aimed to assess the effects of preventative strategies for mastitis and the subsequent effect on breastfeeding duration also showed insufficient evidence as to the effectiveness of a range of interventions [24]. These interventions included education on breastfeeding, changes in breastfeeding habits, hot/cold packs, relaxation techniques, and use of prophylactic antibiotics to prevent recurrence. Only three trials examining the effect of antibiotics were included and although no significant difference in the incidence of mastitis was found, a trend towards a reduced effect was reported (risk ratio (RR) 0.43; 95% confidence interval (CI) 0.11 to 1.61). When one of the trials amongst HIV infected women was excluded, the two remaining studies reported a 67% and 76% reduction in mastitis with antibiotic prophylaxis, respectively [17, 25]. However, all trials were inadequately powered or prematurely stopped due to concerns related to the adverse harms of antibiotic use, issues related to side effects, patient compliance, and attitudes [17, 25]. The review concluded that the use of antibiotics in the absence of clear indications resulted in no benefit and could lead to harm, not just for the participants, but in the production of antibiotic-resistant strains of the organisms. [17, 24, 25] Authors of the Cochrane review recommended future studies should consider implementation of strategies to improve adherence such as extended consultation time with participants when recruiting participants to explain and reinforce instruction; designing interventions such as tailoring drug regimens to patient lifestyle; frequent follow up when initialising or changing treatment regimes; and the use of reminder calls and alerts to keep participants focused [24].

The underlying biological pathogenesis of mastitis is important when considering management options. Mastitis is characterised by the presence of the main aetiological agents of infection, staphylococci and streptococcal organisms. The mastitis causing strains, mainly *staphylococcus aureus* and *streptocococcus epidermidis* usually display two common properties; resistance to antibiotics and a high ability to form biofilms, which may explain their resistance to a wide range of antibiotic therapies and resultant recurrence of disease [26]. A recent WHO report outlines the growing public health concern related to antimicrobial resistance. The report stresses that resistance of common bacteria to antimicrobial drugs has reached alarming levels, with available options for treating infections becoming increasingly ineffective [27]. Further, authors stress the importance of actively seeking safe and effective alternatives to antibiotics.

Probiotics, with their increasing recognition and use in recent years, may provide one such alternative. Probiotics are live microorganisms that when administered in adequate amounts are thought to confer a beneficial effect to the host [28]. Research indicates that specific probiotic bacteria possess significant anti-inflammatory properties comparable to a therapeutic pharmaceutical agent and supports their potential use as immunomodulatory agents [29]. Given the gut microbiota is a critical stimulus for the adequate maturation and function of the immune system [30], oral administration of probiotics to women during the early postnatal period to modulate microbiota composition is thought to provide an effective dietary strategy to reduce the risk of infection and disease [31].

Although the knowledge of the commensal and/or potential probiotic bacteria that exist in milk of healthy women is limited, bacteria commonly isolated from this substrate include *staphylococci, streptococci, micrococci, lactobacilli* and *enterococci*. Of these, *lactobacilli* are considered to constitute the primary microbiological barrier to infection by intestinal and urogenital pathogens. Thus, breast milk of healthy woman may be a source of potentially probiotic or biotherapeutic lactobacilli with a role in protecting mothers and/or infants against infectious diseases. In contrast, mastitis, characterised by inflammation of one or more lobule of the mammary gland, usually has an infectious origin involving *staphylococci, streptococci, and/or corynebacteria,* with staphylococcus aureus thought to be the main aetiological agent of acute mastitis [32]. A number of studies have demonstrated that administration of probiotic bacteria, particularly the lactobacilli strain isolated from human milk, has the potential to prevent and treat maternal breast infections caused by *Staphalycoccus aureus* [26, 32–35]; the key mechanism for this probiotic treatment of mastitis being related to the stimulation of the host intra-mammary immune system. Moreover, findings from a recent randomised trial revealed that two strains of lactobacilli, *Lactobacillus salivarius* and *Lactobacillus fermentum* provided some evidence as to their effectiveness as alternatives to antibiotics for the treatment of mastitis [36]. One of these strains, *Lactobacillus salivarius,* has recently been investigated for the prevention of mastitis [37]. This study, along with previous animal studies have shown promising results [38]. However, due to the limited evidence, there is a need for more thorough investigations of the use of oral probiotics for the prevention of mastitis.

The use of probiotic bacteria during pregnancy and postpartum has also been proposed as a means of modulating maternal infection or inflammatory processes [39] and to support immune development in the infant [31]. Breastfed infants of mothers who consumed specific probiotics in the early postpartum period reported fewer gastrointestinal symptoms [31], while risk of atopic eczema, allergy and asthma was shown to confer some benefit during the first 2 years of life by some studies, but not in others [40]. Further studies with longer follow-up and amongst specific maternal populations most likely to benefit from probiotics, have been recommended. Reassuringly, the probiotic studies that have been performed in postpartum women did not report any major side effects to the mother or infant [39]. Maternal side effects of mild gas or bloating were occasionally noted.

To date, evidence suggests that probiotics may provide an effective measure and alternative to antibiotics in the treatment of lactational mastitis and that postpartum use may also confer additional benefit to the infant. Thus, the efficacy of probiotics in the prevention of mastitis in breastfeeding women should be evaluated.

## Methods/Design

### Aim

The primary aim of the APProve (C**A**n **P**robiotics Im**Prove** Breastfeeding Outcomes?) study is to evaluate the effectiveness of oral probiotics for the prevention of mastitis in breastfeeding women. Secondary aims will assess maternal breastfeeding outcomes, infant infections including gastroenteritis, and the acceptability of a novel mobile phone application system.

### Hypotheses

The primary hypothesis is that regular ingestion of the probiotic *Lactobacillus Fermentum* CECT5716 by women in the first 2 months following birth will decrease the incidence of mastitis in breastfeeding women.

The secondary hypotheses are that exposure to probiotics may also confer a benefit to breastfeeding mothers and reduce risk of infection among infants, particularly gastroenteritis. Furthermore, that the use of a mobile phone application system will provide a novel means of interacting with participants and improve participant compliance, completeness of data collection and reduce recall bias.

### Study design

A double-blind randomised controlled trial will be conducted to assess the outcomes amongst breastfeeding women ingesting a probiotic versus a placebo.

### Setting

The study will be conducted at the Royal North Shore Hospital, Northern Sydney Local Health District (LHD); the Royal Prince Alfred Hospital, Sydney LHD; and the Royal Hospital for Women, South Eastern Sydney LHD; in New South Wales, Australia. The annual number of births at each site are 2600, 5000 and 5000, respectively. Ethics approval for this study has been given by the Northern Sydney Local Health District Human Research Ethics Committee: HREC/14/HAWKE/358 with site specific governance approval provided prior to commencement from each site. The trial is registered with the Australian New Zealand Clinical Trials Registry: ACTRN12615000923561 and conducted according to standardised Good Clinical Practice guidelines.

### Participants/eligibility criteria

#### Inclusion criteria

Women ≥18 years of age who have delivered a singleton baby at 37 weeks’ gestation or later will be invited to participate in the trial. They will currently not be taking commercial probiotics containing *Lactobacillus fermentum*; and will own a smartphone. Their intention at the time of consent will be to breastfeed their baby for at least 2 months following birth.

#### Exclusion criteria

Women with a history of Raynaud syndrome will not be eligible to participate in the trial. Any delivery/breast complication rendering the infant unable to breastfeed will be excluded. Women unable to speak/understand English will not be consented.

### Study procedures

**Fig. 1:**
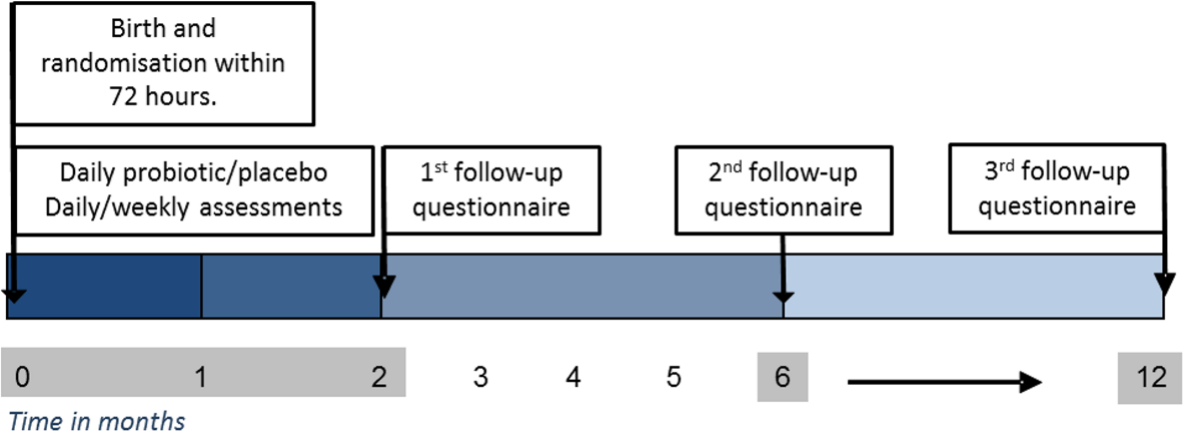
Study schema of procedures and timing of data collection for the APProve trial

The study schema is presented in Fig. 1. Study schema of procedures and timing of data collection for eligible women will be identified and approached by a local researcher in the postnatal ward within 72 h of birth. The researcher will explain the trial, review the Participant Information Sheet, obtain written informed consent and collect baseline data. Women will be reminded that they may withdraw from the trial at any time.

### Randomisation

Randomisation to either “probiotic” or “placebo” will take place immediately after consent within 72 h of birth. The randomisation schedule will be prepared and centrally administered by a researcher not involved in patient care. A computer random number generator will be used to prepare the randomisation schedule in blocks of 4 and 6, and stratified by the incidence of previous mastitis. The randomisation sequence will be concealed until all data has been collected. The participant and researcher will be blinded as to treatment allocation. Participants using the mobile phone application system (APProve-Lite) will be randomised via a central password-protected web-based application developed by the APProve clinical trial unit. Concealment for participants using the ‘standard’ approach (not the APProve-Lite system) will be via opaque, sealed envelopes.

### Intervention

The participant will be given an 8 week supply of her allocated treatment. The probiotic sachets and placebo sachets will be identical in every respect except for the *Lactobacillus Fermentum* CECT5716 (1 × 10^10^ CFU/mL) ingredient contained in the probiotic. Participants will be advised to take one sachet daily, preferably at the same time each day for a period of 8 weeks following the birth of her baby. As it is recommended to store the product below 25 °C, participants will be asked to refrigerate the boxes of sachets at home. The contents of the sachet should be mixed with water, juice or milk, stirred and consumed immediately. Women will be advised not to make up for missed doses by double-dosing, but rather to continue their daily routine as soon as possible. The number of unused sachets will be collected by the research coordinator at the end of the 8-week period.

Participants will be encouraged to maintain routine health care. If antibiotics are prescribed, women will be encouraged to continue with their treatment regime, but advised to take the treatment sachet at least 2 h after taking the antibiotic.

Participants using the APProve-Lite system (APP) will receive daily questionnaires collecting data on treatment intake, breast pain, infection symptoms and infant feeding. Weekly questionnaires will collect data on maternal and infant well-being, unscheduled doctor’s visits, and medication intake. Women not using APProve-Lite will receive an 8-week calendar diary to record their daily responses, along with a weekly questionnaire accessed by an emailed web-link.

### Follow-up

Follow-up assessments will be conducted at 2, 6 and 12 months postpartum via postal questionnaire, email web-link, or the study APP depending on the participant’s preference. Non-APP participants will receive the questionnaire in a web-link sent by email. The questionnaires will be self-completed and assess general maternal and infant well-being, stress levels, hospital/doctor visits, recurrent symptoms, and maternal/infant feeding practices, and will utilize validated patient surveys including the SF-12 Quality of Life Questionnaire [41] and the State-Trait Anxiety Inventory (STAI-6) [42].

### Outcome measures

The primary outcome will be defined as the incidence of mastitis up to 8 weeks following birth as measured by:

1. Clinical diagnosis of mastitis OR
2. At least two of the following breast symptoms: pain, redness/inflammation, lump/swelling AND at least one of the following systemic symptoms: flu-like symptoms (body aches, headaches and chills) or fever ≥38°C. These symptoms must be present for at least 24 h [43].

Secondary maternal outcomes will include breastfeeding duration (total/partial), recurrence of mastitis, development of breast abscess, cracked nipples, use of antibiotics, overall maternal health and well-being, breastfeeding support, number of doctor’s visits for probable mastitis, overall doctor’s visits, adverse effects of treatment, and incidence of primary mastitis between 2 and 6 months postpartum. Maternal lifestyle factors which may affect breastfeeding outcomes will also be assessed. Acceptability and compliance of the trial product and the APProve-Lite system will also be examined as well as preference for method of postnatal questionnaires.

Secondary infant outcomes will include growth (height and weight) and well-being in the first year of life (measured at 2, 6 and 12 months as assessed via self-report of health conditions including infections (gastrointestinal, respiratory), doctor’s visits, admission to hospital, allergic reactions and/or use of antibiotics.

### Data collection

To assess the comparability of the study groups, baseline demographic and medical information will be collected at the time of entry into the study. The baseline assessment will include maternal age, socio-demographic data, smoking and alcohol use, infections during pregnancy, allergies, use of vitamins/supplements/probiotics in the previous month, history of prior pregnancies and lactations including previous mastitis and breastfeeding duration, and recent use of antibiotics. This information will be obtained from the participant and/or her medical record.

At the time of discharge, data related to the birth and infant feeding will be collected to evaluate the impact of events and outcomes around the time of birth on infant feeding practices. This information will be ascertained from both the participant and medical records and will include site, gestation at birth, mode of birth, antibiotics during labour, birth or postpartum, complications of birth, length of hospital stay and initiation of infant feeding. Baseline infant data on general characteristics and well-being will also be collected. This will include information on infant sex, birthweight, Apgar scores at 1 and 5 min, resuscitation and any morbidity and will be obtained from the infant’s medical record.

Follow-up questionnaires will be used to evaluate long term maternal and infant secondary outcomes as detailed above with timing and method of collected data presented in Table 1.

**Table 1:** Timing of data collection for the APProve trial

Peripartum Period	Intervention	Timing
Birth (1–72 h)	Identification of eligible women	≥37 weeks’ gestation
	Patient information given, consent, baseline data collected	

Postnatal (Birth to 8 weeks)	Data collection of treatment compliance, breast pain, infection symptoms and infant feeding	Daily x 56 (APP/calendar diary)
	Data collection of well-being, Drs’ visits, and medication intake	Weekly x 8 (APP/email)
Postnatal (2,6 & 12 months)	Follow up questionnaire (post, email or APP)	2 months postpartum
	Follow up questionnaire (post, email or APP)	6 months postpartum
	Final follow up questionnaire (post, email or APP)	12 months postpartum

### Independent assessment of intervention and mobile phone application system

A separate sub-study will be conducted to assess the independent effect of the probiotic without the potential influence of the APProve-Lite system. Despite the APP being a tool to facilitate data collection and improve compliance, it may be viewed as an intervention in itself, and have an unintended effect on study outcomes. Assessment of the APP is important to ensure this tool does not bias or exert any influence on study outcomes. This will involve a sub-study utilising a pragmatic and ‘standard follow-up’ approach to data collection and follow-up of women throughout the trial for collection of information on symptoms related to study outcomes and ensuring compliance with the ingestion of the intervention.

The sub-study will involve a sample of 100 women who will be recruited on randomly selected days of each month. These women will be recruited and enrolled into the trial in an identical way to those of the APP group and randomised to the intervention as in the main study. However, they will not be asked to use the APP throughout the trial. Rather, these women will be provided with a 2-month calendar diary to complete each day. They will be encouraged to place this in a visible location such as on the fridge to encourage daily compliance. Here they will record daily ingestion of the probiotic and symptoms via check boxes (daily survey). They will be provided with an addressed reply-paid envelope in which to post the completed diary back to the recruiting center. Each week, women will be emailed a link to an online Survey Monkey questionnaire to complete (weekly survey). At the end of the intervention, women will be emailed a web-link to the 2, 6 and 12-month follow-up questionnaires.

### Data monitoring committee

The data monitoring committee (DMC) will be established to monitor the trial and review the study progress. The DMC will meet once half way through trial recruitment to review the safety data and monitor the progress of the trial. There will be only one analysis at the end of the trial. The Committee members will be independent of the trial, free of conflicts with any of the investigative team. The terms of reference and functions are derived from the principles established by the Data and Safety Monitoring Boards: Lessons, Ethics, Statistics Study Group charter. The DMC will report its recommendations directly to the Trial Steering Committee, who are the authors of this protocol.

### Adverse event reporting

#### Adverse event

Side effects of the probiotic will be collected via the 2-month questionnaire. Mild gas and bloating are the only known reported side effects of *Lactobacillus fermentum*. Reported incidents will be reported to the DMC for review.

#### Serious adverse event

Any maternal or infant deaths will be reported to the ethics committee as required and reviewed by the DMC.

#### Suspected unsuspected serious adverse reaction (SUSAR)

We do not anticipate any SUSARs in relation to this trial.

### Sample size

Given the lack of evidence related to the potential effect of probiotic use for the prevention of mastitis, we used the results from the comparable trials of antibiotics for the prevention of mastitis to determine effect sizes. Although the Cochrane review reported an overall 57% non-significant reduction in mastitis [24], with individual trials finding an up to 76% reduced risk [25], we chose a more conservative 50% reduction as a clinically meaningful treatment effect. Based on an expected rate of mastitis in the control group of 18% [9–11], two-sided 5% significance level and a power of 80%, we estimated a total sample size of 452 would be required. However, given studies related to the duration of breastfeeding report a cessation rate in the first 8 weeks post-partum of up to 20% [9, 15], and a potential loss-to follow-up of 5–10% may occur with withdrawals or non-compliance, we inflated the sample size by a further 30% to ensure complete data. Hence, we estimated a total sample size of approximately 600 women would be required (300 per group) for this trial, including all sites.

### Statistical analyses

The analysis and reporting of the results will follow the CONSORT guidelines (http://www.consort-statement.org/). Primary and secondary outcomes will be analysed using an intention-to-treat approach, which includes all losses-to follow-up, non-compliances or withdrawals. Baseline characteristics will be compared between allocated treatment groups using frequencies and descriptive statistics. The principal analysis will use an unadjusted Chi-Square test to compare the incidence of mastitis between the two groups. The corresponding unadjusted relative risk with 95% confidence interval will also be calculated. Further analysis of the primary outcome will use multivariable logistic regression to examine the effect of adjusting for any imbalances at baseline or important predictors of mastitis (e.g. previous history of mastitis, antibiotic use, cracked nipples, or duration of breastfeeding). Additional unadjusted and adjusted analyses of all secondary outcomes will be performed using measures and multivariable models appropriate for the outcome. An analysis of women compliant with treatment (per protocol) will also be performed to assess the robustness of the primary findings. For all analyses, the number of cases with complete data and those with missing values will be reported. If necessary, reason for and type of missing data will be assessed to determine whether multiple imputation, sensitivity analysis or other approaches (“best” and “worst” case scenario, last value carried forward) will be required. Analyses will be performed using SPSS Version 24 (IBM SPSS Statistics, 2016 IBM Corporation, Chicago, IL) by an analyst not involved in any data collection and blinded to treatment allocation.

To assess the impact of the APP, the rate of primary and secondary study outcomes will be compared between the main study group and small sub-sample. Compliance rates in the ingestion of the intervention, and completeness and acceptability of data collected during daily and weekly follow-up will also be examined between the two study groups.

This protocol has been formatted according to the SPIRIT (Standard Protocol Items: Recommendations for Interventional Trials) guidelines (Additional file 1).

## Discussion

Interventions that support prolonging breastfeeding duration have the potential to improve human health. Mastitis is a painful and debilitating complication of breastfeeding which affects 1 in 5 breastfeeding mothers. It is associated with early cessation of breastfeeding and, if severe, may lead to unwarranted hospital admissions for treatment of abscess often requiring treatment with antibiotics. At a time when we are experiencing the negative effects of antibiotic overuse, there is an urgent need to explore safe and effective alternatives for preventing infections such as mastitis in lactating women. This trial seeks to provide evidence for such an alternative in the form of probiotics, which may increase breastfeeding duration and potentially provide long-term health, cognitive and developmental benefits for children, as well as decrease antibiotic usage with benefits to the community and health system.

## Additional file

Additional file 1: SPIRIT (Standard Protocol Items: Recommendations for Interventional Trials) guidelines. (DOC 120 kb)

## Abbreviations

AIHW: Australian Institute of Health and Welfare
APP: APProve-Lite system
CI: Confidence interval
DMC: Data Monitoring Committee
LHD: Local health district
RR: Risk ratio
SUSAR: Suspected unsuspected serious adverse reaction
WHO: World Health Organisation
